# Scroll Ligament Repair in Dorsal Preservation Rhinoplasty: A Cartilage as a Guide on Nasal Skin Envelope for Suturing

**DOI:** 10.1007/s00266-025-04717-y

**Published:** 2025-02-18

**Authors:** Yavuz Tuluy, Vasfi Çelik, Aziz Parspancı, Emin Sır

**Affiliations:** 1 Department of Plastic, Reconstructive and Aesthetic Surgery, İzmir Bakırçay University Çiğli Training and Research Hospital, İzmir, Türkiye; 2 Private Practice, Plastic, Reconstructive and Aesthetic Surgery, Mersin, Türkiye; 3 Private Practice, Plastic, Reconstructive and Aesthetic Surgery, İstanbul, Türkiye; 4 Department of Plastic, Reconstructive and Aesthetic Surgery, İzmir Kavram Vocational School, İzmir, Türkiye

**Keywords:** Scroll ligament, Preservation rhinoplasty, Functional rhinoplasty, Nasal skin envelope, Patient satisfaction

## Abstract

**Background:**

The scroll ligament provides support to the internal and external valve, and its repair in rhinoplasty provides good functional and aesthetic results. In this study, it was aimed to repair the scroll ligament complex by leaving cartilage in the nasal skin envelope, and clinical results were shared.

**Patients and Methods:**

216 patients who underwent closed preservation rhinoplasty with modified low septal strip septoplasty were included in the study. A 10 x 1 mm of cartilage is left in the nasal skin envelope at the midpoint of the cranial part of the lateral crus of the LLC, leaving it as a guide to repair the scroll ligament complex in the anatomically correct place. Demographic data, complications, revision surgeries, follow-up periods and satisfaction of the patients were analyzed retrospectively.

**Results:**

Residual humps were observed in 2 patients. Inferior turbinate hypertrophy was observed in 4 patients. In 1 patient, total septal reconstruction was performed. Two hundred and three patients were evaluated the results as poor, moderate, good and very good. One hundred and sixteen patients rated the functional outcome as very good, 80 patients as good, 4 patients as moderate and 3 patients as poor. One hundred and twenty-eight patients rated the aesthetic result as very good, 72 as good and 3 as moderate.

**Conclusion:**

Repairing the scroll ligament complex provides both internal and external valve support, better redraping and eliminating the dead space. Leaving cartilage on the nasal skin envelope as a guide helps to repair the scroll ligament complex in the anatomically correct place at the end of the surgery.

**Level of Evidence IV:**

This journal requires that authors assign a level of evidence to each article. For a full description of these Evidence-Based Medicine ratings, please refer to the Table of Contents or the online Instructions to Authors www.springer.com/00266.

**Supplementary Information:**

The online version contains supplementary material available at 10.1007/s00266-025-04717-y.

## Introduction

Rhinoplasty is one of the most performed aesthetic operations in the world, and the aim of this surgery is not only aesthetic, but also to achieve a good functional result. A nose that looks very beautiful but cannot breathe is the result of a failed surgery, and the primary consideration in rhinoplasty surgery should be to preserve or improve the functional properties of the nose. What should be done to preserve the functional properties of the nose is not to narrow the nasal width too much with osteotomies and to preserve the volume, to perform an effective septoplasty, to preserve the internal valve angle, to provide adequate rotation in the nasal tip, to correct the deviations and to preserve the scroll ligament. Most of these are taken into consideration, but scroll ligament repair or preservation is ignored in most cases. In a histologic study performed to better understand the scroll ligament, it was observed that the fibers were parallel, oblique and perpendicular to the cartilages^1^. The scroll ligament, which has transverse and vertical components, provides a connection between the lower lateral cartilage (LLC), the upper lateral cartilage (ULC) and the nasal skin envelope (NSE), preventing collapse of this area^2^. It supports internal and external nasal valves^3^. Repairing the scroll ligament in rhinoplasty surgery is important for this reason. However, when placing this suture, its localization must be adjusted correctly. Thus, it is ensured that the dead space that will occur if it cannot be repaired is partially eliminated without creating a dimple from the outside. In this study, we aimed to create a guide for suturing by leaving a 10×1 mm cartilage in the NSE while dissecting the lateral crus of the LLC in patients undergoing dorsal preservation rhinoplasty (DPR) and shared our clinical results.

## Patients and Methods

Two hundred and sixteen patients who underwent closed DPR with low septal strip septoplasty between November 2021 and January 2024 were included in the study. All the cases were primary rhinoplasty, and revision rhinoplasty cases were not included in the study. Patients were discharged on the first postoperative day. Oral antibiotics and analgesics were given for the first week. Nasal splints and silicone nose pads were removed on the 5^th^ postoperative day. Patients were asked to evaluate the results separately in terms of aesthetics and function. The results were evaluated as poor, moderate, good and very good. Patients were contacted at their last follow-up examination or by telephone, and data were collected. This research was conducted as per the Declaration of Helsinki guidelines. All the patients have provided written informed consent for the surgery and use of their data. Demographic data, complications, revision surgeries, follow-up periods and satisfaction of the patients were analyzed retrospectively.

## Surgical Technique

**Fig. 1:**
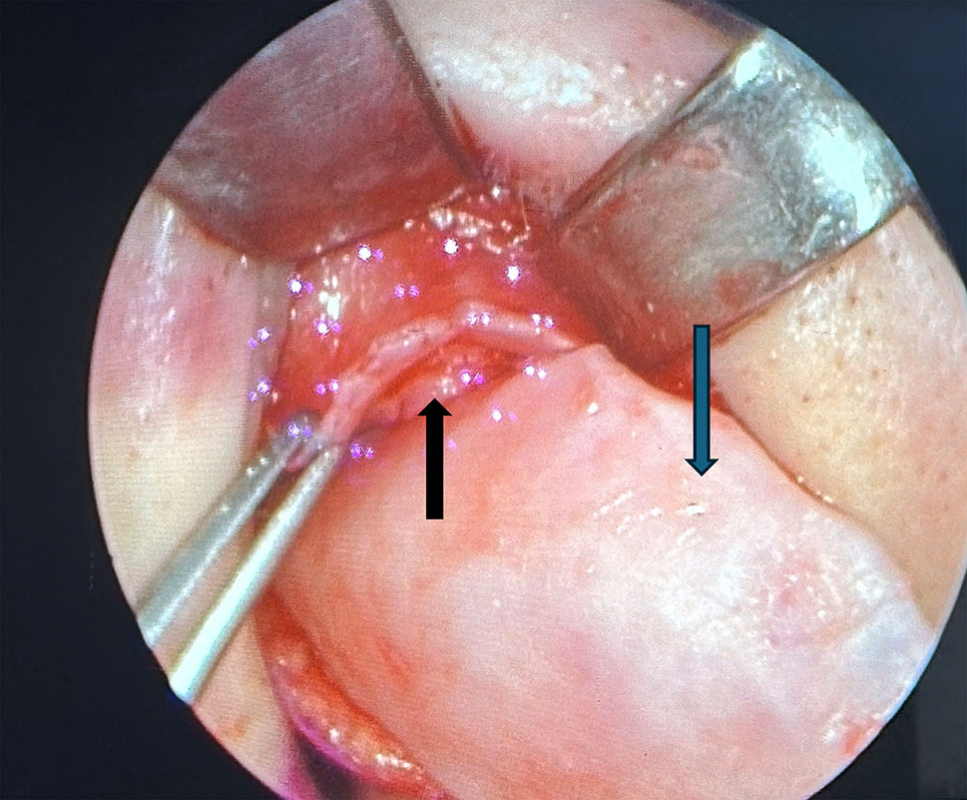
Perioperative view of the ULC, LLC and cartilage as a guide left attached to the NSE. Forceps: cartilage as a guide on NSE, blue arrow: ULC, black arrow: LLC

**Fig. 2:**
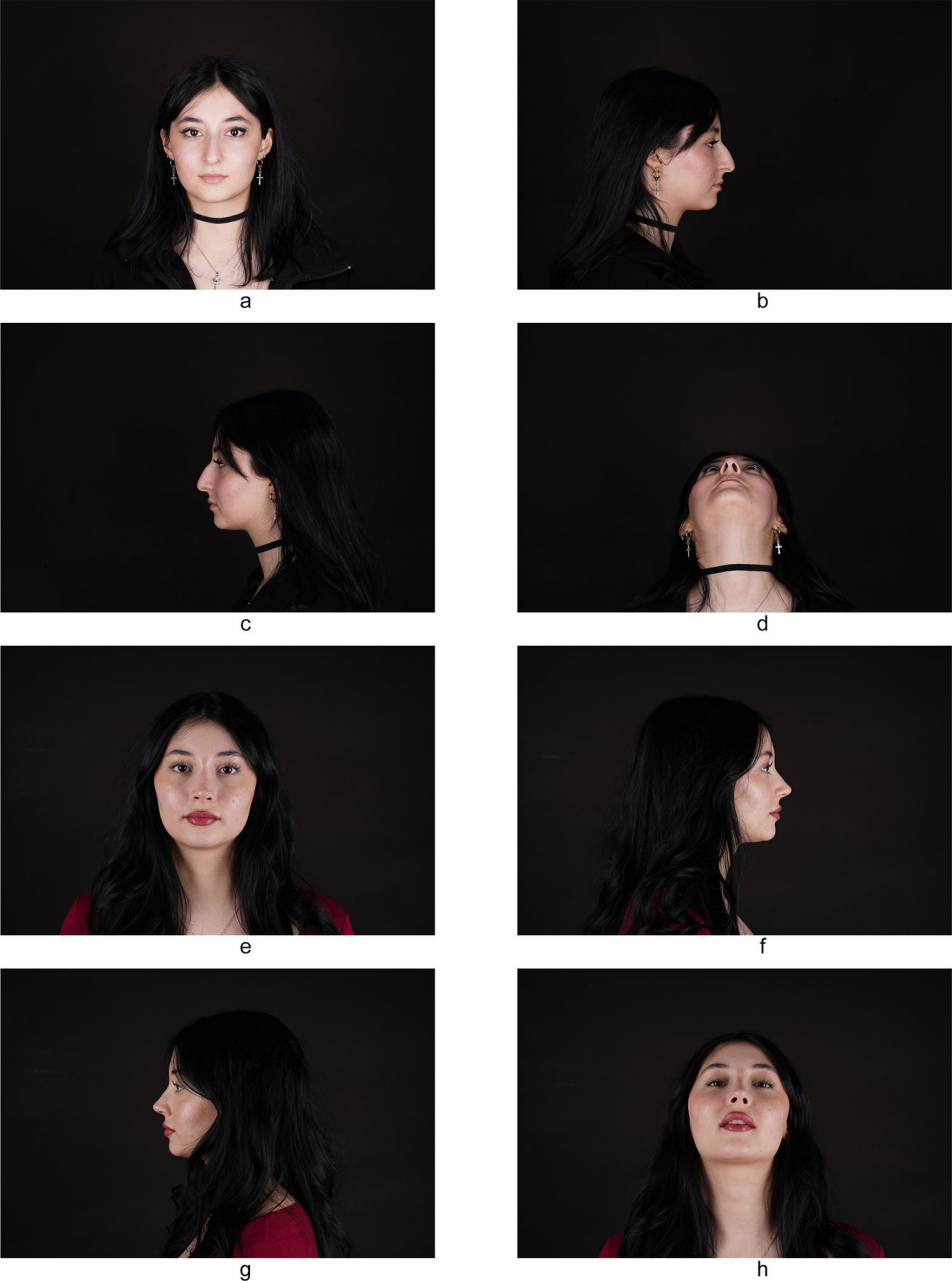
**a-d** Preoperative view of a 22-year-old patient and **e-h** postoperative view of the patient

**Fig. 3:**
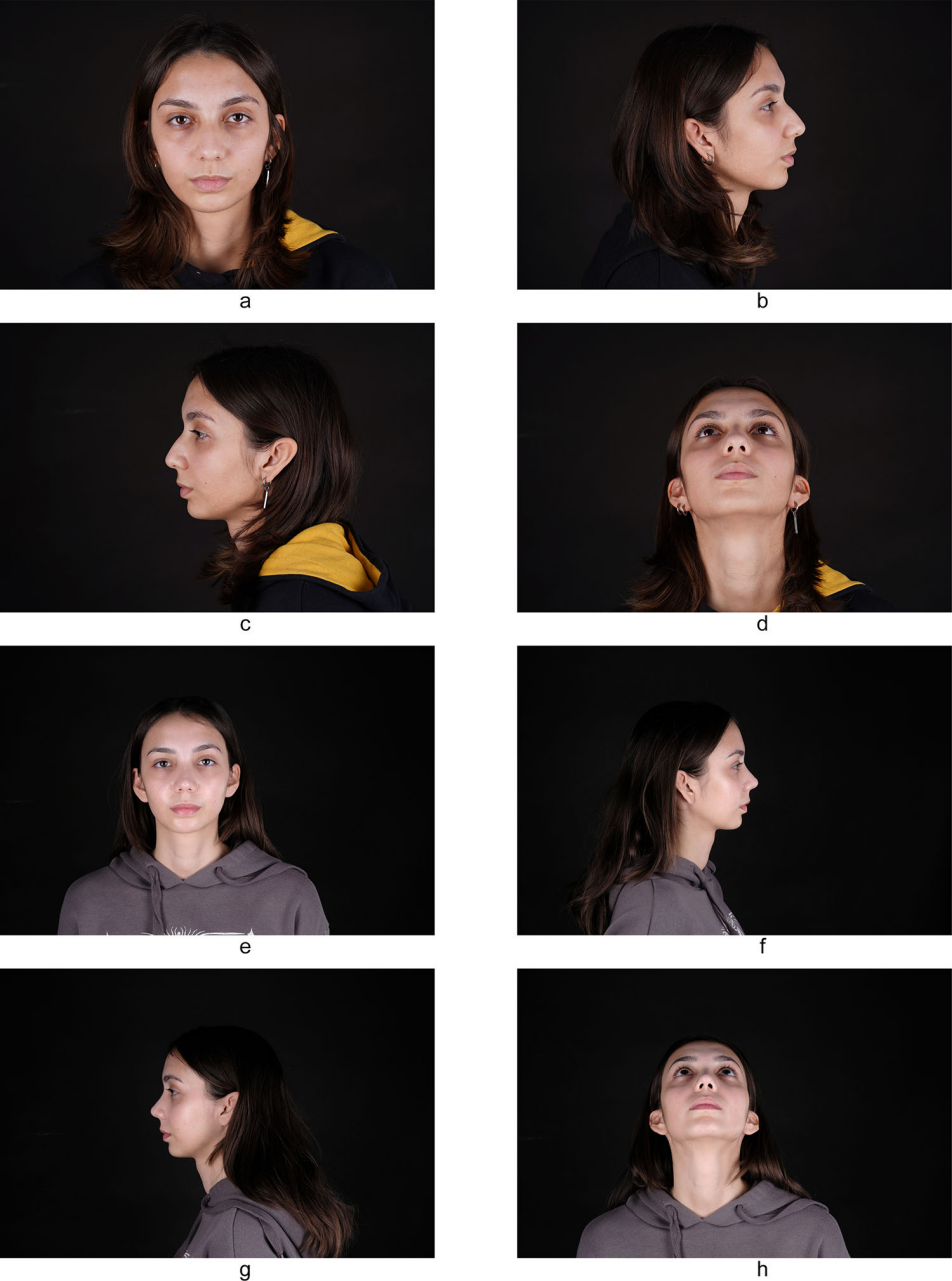
**a-d** Preoperative view of a 24-year-old patient and **e-h** postoperative view of the patient

Closed dorsal preservation with low septal strip septoplasty technique was performed under general anesthesia with systolic blood pressure maintained at 80–90 mm Hg. Following local anesthetic infiltration, infracartilaginous and hemitransfixation incisions were performed, and the septum, ULCs and LLCs were exposed in the supraperichondreal plane. After the infracartilaginous incision is made, the dissection continues cranially with the help of an elevator. When the scroll ligament located at the junction of the ULC and lateral crus of LLC is reached, two important components of this ligament are seen. The first is the vertical scroll ligament that provides the connection between the nasal skin and the cartilages, and the second is the transverse scroll ligament that provides the connection between the ULC and lateral crus of LLC. At this point, approximately 10x1 mm of cartilage is left in the nasal skin envelope at the midpoint of the cranial part of the lateral crus of the LLC, leaving it as a guide to repair the vertical scroll ligament in the anatomically correct place at the end of the surgery **(**Fig 1**, **Video 1**)**. After septoplasty and osteotomies are performed and the dorsum is corrected, tip surgery is started. We use triangular bilateral septal extension grafts caudal to the septum and thus ensure tip projection and rotation not only with sutures, but also with the support of cartilage grafts. Some patients have excess cartilage caudal to the ULC after achieving type rotation. When this excess cartilage is not resected, it disrupts the tip rotation and causes a concave appearance in the ULC. In these patients, the excess is usually 1–2 mm and after resecting these, we proceed to scroll ligament complex repair. After cephalic resection or slide under of the LLC, the suture is passed through the cranial part of the middle part of the LLC with 5/0 PDS, the suture is passed through the ULC and then the suture is completed by passing through the cartilage left as a guide in the NSE **(Video 2–3)**. Clinical results of the patients are shown in Fig2a-h, 3a-h and 3d animation of the technique is presented in Video 4.

## Results

The surgeries were performed under general anesthesia. 73.9% (n=162) of the patients were female and 26.1% (n=57) were male. The mean age of the patients is 27.5(17–65). The mean follow-up period was 12.5 months. Patients were discharged on the first postoperative day. Oral antibiotics and analgesics were given for the first week. Nasal splints and silicone nose pads were removed on the 5^th^ postoperative day. Residual humps were observed in 2 patients, and dorsum rasping was performed under local anesthesia. In 4 patients with a history of allergic rhinitis before surgery, inferior turbinate hypertrophy was observed during follow-up, and radiofrequency was performed. In 1 patient, total septal reconstruction was performed during primary rhinoplasty due to severe deviation and disruption of the structure secondary to previous trauma. Thirteen patients could not be reached by phone or were not evaluated the results. One hundred and sixteen patients rated the functional outcome as very good, 80 patients as good, 4 patients as moderate and 3 patients as poor. One hundred and twenty-eight patients rated the aesthetic result as very good, 72 as good and 3 as moderate.

## Discussion

Preservation or repair of the scroll ligament complex is important to achieve good functional results. It supports internal and external nasal valves and provides functional improvement^3^. Repair of the scroll ligament not only contributes to the airway, but also prevents pinch deformity. After the importance of this ligament was demonstrated by clinical and histologic studies, interest has increased and new techniques for its reconstruction have been defined^4,5^. It was observed that the scroll ligament had longitudinal and vertical fibrous attachments, and this ligament was named as scroll ligament complex^6,7^.

In a study conducted by Özmen et al. in 2009, the sliding alar cartilage flap technique was described^8^. In the study, in patients who underwent open rhinoplasty, after preserving the caudal 6 mm part of the LLC, the cephalic part was incised without cartilage resection, and a flap was created by preserving the scroll ligament. This flap was advanced and sutured after creating a pocket between the caudal LLC and the mucosa. In this technique, while the longitudinal fibers of the scroll ligament are preserved, the vertical fibers are not. In a study by Öztürk, an incision is made along the lateral crus, preserving the caudal 6 mm of the crus. After resecting excess cartilage from the cartilage left intact with the scroll ligament on the cranial side, a superior-based sliding flap is prepared. Then, a pocket is prepared by undermining the caudal part of LLC and mucosa, and the 3 mm superiorly based sliding flap is inset to the pocket^9^. In this technique, both vertical and longitudinal scroll ligaments are preserved because dissections are performed from two different rooms. Successful results have been obtained both functionally and aesthetically. In the study by Taş, after dissecting the lateral crus in the subperichondrial plane, a 6-mm wide lateral crus is preserved and incision was performed along the caudal course of the crus, and a superiorly based flap was designed and sutured over the LLC^10^. In this study, the longitudinal and vertical fibers of the scroll ligament are preserved. In a study by Bitik et al, the deep lateral portion of the nasal SMAS is tagged with suture and then cut^3^. This maneuver makes it easier to find the correct anatomical location due to retraction of the soft tissue during the scroll ligament. After performing the necessary resections in LLCs and ULCs, the scroll ligament was repaired. In the study by Tellioğlu and Çimen, they described the turn-in folding technique to correct the concavity in the lateral crus of the LLC and prevent collapse of the lateral crus^11^. In this study, after preserving the 6-mm lateral crus, the cephalic part was disconnected from the ULC and sutured by turning it under the lateral crus. In this technique, the lateral crus is strengthened and convexity, if present, is corrected, but the scroll ligament is not repaired.

In our study, we performed suturing by leaving cartilage in the NSE to reconstruct the scroll ligament complex in the correct localization to restore the normal anatomy. In our study, this technique was applied in closed rhinoplasty and can be easily applied in open rhinoplasty. Repairing the scroll ligament complex provides both internal and external valve support, better redraping and eliminates the dead space. Since it contributes to redraping, it prevents the formation of dead space and prevents the fullness of this area. The cartilage left in the skin envelope is a 10x1 mm piece of cartilage and acts as a cephalic continuation of the lateral crus. Therefore, it does not cause a negative change in the tip position. Since 3-point fixation is performed, there is no problem in the alignment of the cartilage that will reflect externally or change the tip position. Thus, it provides aesthetically and functionally positive results. The results were evaluated aesthetically and functionally by the patients. In the study, good aesthetic and functional results were obtained in the majority of patients. The cartilage left as a guide during scroll ligament complex repair was not resected, and this did not lead to an irregularity in the external appearance. However, if desired, it can be resected after suturing. This allows our technique to be modified according to the surgeon's choices. It allows the surgeon to make the desired resections from the ULC and LLC and allows it to be applied not only in DPR, but also in structural rhinoplasty. The limitations of our study are that it was retrospective, and there was no control group. Another limitation of the study is that no validated questionnaire was used.

## Conclusion

Repairing the scroll ligament complex provides both internal and external valve support, better redraping and eliminates the dead space. It provides aesthetically and functionally positive results. Leaving cartilage on the nasal skin envelope as a guide helps to repair the scroll ligament complex in the anatomically correct place at the end of the surgery.

## Supplementary Information

Below is the link to the electronic supplementary material.

Supplementary file1 (MP4 17194 KB)

Supplementary file2 (MP4 4955 KB)

Supplementary file3 (MP4 44912 KB)

Supplementary file4 (MP4 2361 KB)
